# An Imaging Plane Calibration Method for MIMO Radar Imaging

**DOI:** 10.3390/s19235261

**Published:** 2019-11-29

**Authors:** Yuanyue Guo, Bo Yuan, Zhaohui Wang, Rui Xia

**Affiliations:** Key Laboratory of Electromagnetic Space Information, Chinese Academy of Sciences, University of Science and Technology of China, Hefei 230026, China; yuanb@mail.ustc.edu.cn (B.Y.); wzh01@mail.ustc.edu.cn (Z.W.); xrke928@mail.ustc.edu.cn (R.X.)

**Keywords:** two-dimensional radar imaging, multiple-input multiple-output (MIMO) radar, Particle Swarm Optimization (PSO), imaging plane calibration algorithm (IPCA)

## Abstract

In two dimensional cross-range multiple-input multiple-output radar imaging for aerial targets, due to the non-cooperative movement of the targets, the estimated imaging plane parameters, namely the center and the posture angles of the imaging plane, may have deviations from true values, which defocus the final image. This problem is called imaging plane mismatch in this paper. Focusing on this problem, firstly the deviations of spatial spectrum fulfilling region caused by imaging plane mismatch is analyzed, as well as the errors of the corresponding spatial spectral values. Thereupon, the calibration operation is deduced when the imaging plane parameters are accurately obtained. Afterwards, an imaging plane calibration algorithm is proposed to utilize particle swarm optimization to search out the imaging plane parameters. Finally, it is demonstrated through simulations that the proposed algorithm can accurately estimate the imaging plane parameters and achieve good image focusing performance.

## 1. Introduction

Aerial targets imaging is an important research direction in the field of radar imaging technology. Especially, it plays a crucial role in the military field, such as, aerial defense [1] and anti-missile defense [2] and so on. Multiple-input multiple-output (MIMO) radar is a new radar technique, which adopts multiple transmitters and receivers. By transmitting orthogonal space–time block codes [3] or frequency diversity signals [4], a MIMO radar with *M* transmitters and *N* receivers can eventually form a virtual array of the aperture length up to *M* times that of the receive array, which greatly saves the hardware cost. In addition, MIMO radar can obtain the images of the aerial targets with only one snapshot, and thus has enormous superiority in radar image acquisition time [5,6].

Since MIMO radar technique was proposed, it has been desired to build high performance imaging algorithms. The researches of MIMO radar imaging algorithms mainly focus on two aspects: The first is the wave-number domain imaging methods, and their imaging performances mainly depend on the spatial spectrum fulfilling region, which is generally required to be uniformly fulfilled, so that the fast Fourier transform (FFT) can be applied [7,8]. In this regard, Prof. Yarovoy et al. have conducted a large number of studies and verified the feasibility of the proposed algorithms in security check [9], wall penetrating [10] and ground penetrating imaging applications [7]. In addition to the traditional wave-number domain methods, the iterative optimization methods have also been applied in the MIMO radar imaging field. For example, Prof. Li’s team of University of Florida proposed several iterative imaging algorithms, such as iterative adaptive approaches (IAA) algorithm [11] and the sparse learning via iterative minimization (SLIM) [12] algorithm, which have been well applied and verified in MIMO radar scenarios.

In MIMO radar imaging, the model mismatch caused by system errors or array spatial position errors will degrade the imaging quality. Therefore, the study of model error calibration algorithms is an important research direction of high-quality MIMO radar imaging. In this regard, a large number of studies have been used to calibrate the phase error [13,14], carrier frequency deviation [15], array position error [16], off-grid problem [17,18], and so on. The degradation of MIMO radar resolution under the condition of phase error from the perspective of point spread function (PSF) is analyzed in [13], and the sparse imaging via expectation maximization (SIEM) algorithm is proposed, which alternately estimates the phase errors and the target image, and obtains better imaging quality. Subsequently, the degradation of MIMO radar resolution under the condition of carrier frequency deviation from the perspective of PSF is analyzed in [15] as well, and an iterative algorithm employing iteration strategy similar to reference [13] is proposed, and good imaging results are achieved. MIMO imaging with array position errors is studied in reference [16], while in reference [17,18], the off-grid problem of MIMO radar imaging is studied. The algorithms proposed in [16,17,18] all employ sparse optimization by alternately estimating the target image and the errors during iterations, hence clear images are finally obtained.

Most of the above methods are proposed for two dimensional (2D) imaging in range and cross-range directions. However, for the target plane parallel to cross-range direction, these methods cannot be directly applied. In fact, it is necessary to set the origin of the coordinates at the center of the imaging plane [5,19] for the 2D cross-range imaging methods based on the spatial spectrum. In practical applications, as the target is non-cooperative, it is required to estimate the scene center and posture angles of the target plane, so as to make the final image focus on the image scene center and the target plane. However, there are always some deviations between the estimated imaging plane parameters and the real situations, which resulting in unfocused image and poor imaging quality. This problem is called the imaging plane mismatch problem in this paper.

To solve this problem, firstly, the deviations of spatial spectral fulfilling region caused by imaging plane mismatch are analyzed, and the location errors between estimated spatial spectral point and real spatial spectral point under the condition of imaging plane mismatch are deduced, as well as the errors of the corresponding spatial spectral values. Subsequently, in order to estimate imaging plane parameters and to be able to calibrate the locations and values of spatial spectral points, an imaging plane calibration algorithm (IPCA) is proposed. Aiming at minimizing the image entropy as well as promoting target sparsity, IPCA utilizes a particle swarm optimization (PSO) [20,21] algorithm to search out the parameters of imaging plane center deviation and pose angles deviations, and then calibrates the locations of spatial spectral points according to these parameters, so as to obtain images with better quality.

This paper is organized as follows. Section 2 introduces the spatial spectral imaging model of MIMO radar, and analyzes the problem of imaging plane mismatch, and the deviations between the estimated spatial spectral point and the real spatial spectral point position under the imaging plane mismatch are deduced. Section 3 provides the design and the detailed flow of IPCA. In Section 4, the validity of the proposed algorithm, robustness to noise, and tolerance to mismatching parameters are verified by simulations. Section 5 is the conclusion of this paper.

## 2. Problem Formulation of Imaging Plane Mismatch

In this section, the spatial spectral imaging model of MIMO radar is reviewed firstly, and then the model mismatch problem caused by the imaging plane mismatch is analyzed. Under the condition of imaging plane mismatch, the deviations between the positions of the obtained spatial spectral points and the positions of the real spectral points are analyzed, as well as the values of the corresponding spatial spectral points. Afterwards, the calibration operation is deduced to obtain the focused image after obtaining the imaging plane parameters. Notice that the radar system discussed here is the frequency diversity MIMO (f-MIMO) radar [4,22].

### 2.1. Space Spectral Imaging Model of Multiple-Input Multiple-Output (MIMO) Radar

In this subsection, the spatial spectral imaging model of MIMO radar is reviewed firstly.

**Figure 1:**
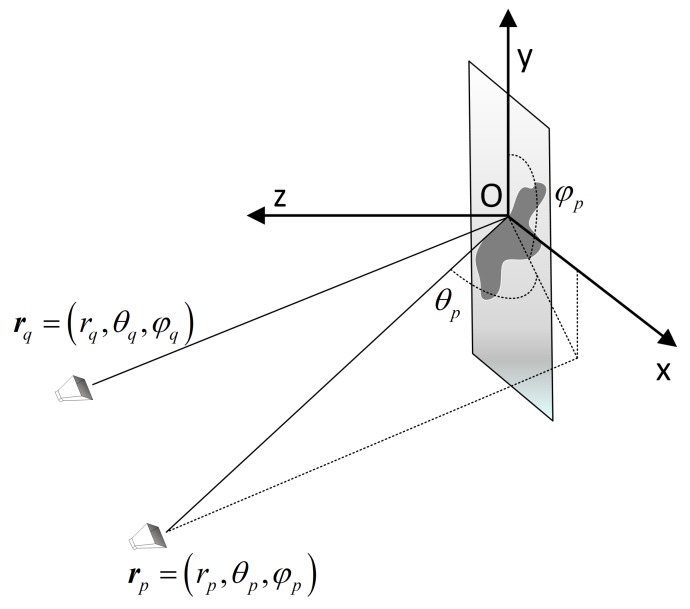
Space spectral imaging model of multiple-input multiple-output (MIMO) radar.

Figure 1 illustrates the general 2D cross-range imaging scenario of MIMO radar. Let (x,y,z) be Cartesian coordinates with the origin *O* located at the center of the imaging plane and the 2D target is supposed to be on the imaging plane. The location of the *p*-th transmitting antenna and the *q*-th receiving antenna are denoted as $r_{p}=\left(r_{p},\theta_{p},\varphi_{p}\right)$ and $r_{q}=\left(r_{q},\theta_{q},\varphi_{q}\right)$ in spherical coordinate respectively.

Without loss of generality, regardless of the loss associated with the free-space propagation, the echo signal at the *q*-th receiver by the *p*-th transmitter is given by [5]:

(1) $$s_{p,q}\left(t\right)=\;\int \int \;\sigma \left(x_{T},y_{T}\right)\exp\left(j2\pi \left[f_{p}\left(t-\frac{R_{p,T}}{c}-\frac{R_{q,T}}{c}\right)\right]\right)dx_{T}dy_{T}$$

where $\sigma (x_{T},y_{T})$ denotes the reflectivity of the scatterer at $\left(x_{T},y_{T}\right)$ on the imaging plane, $f_{p}$ is the transmitting frequency of the *p*-th transmitter and *c* is the speed of light. $R_{p,T}=\left|r_{p}-r_{T}\right|$, $\;R_{q,T}=\left|r_{q}-r_{T}\right|$, $r_{T}=\left(x_{T},y_{T},0\right)$.

Then, down conversion is applied to the received signal, which can be achieved by multiplying it by the following reference signal:

(2) $$s_{ref}\left(t\right)=\exp\left(-j2\pi \left[f_{p}\left(t-\frac{\left|r_{p}\right|}{c}-\frac{\left|r_{q}\right|}{c}\right)\right]\right)$$

In the case of the far field, approximate conditions can be used:

(3) $$\begin{array}{cc} R_{p,T} & =\left|r_{p}-r_{T}\right|=\left|r_{p}\right|-r_{T}\cdot {\hat{e}}_{p}\\ R_{q,T} & =\left|r_{q}-r_{T}\right|=\left|r_{q}\right|-r_{T}\cdot {\hat{e}}_{q} \end{array}$$

where ${\hat{e}}_{p}=r_{p}/\left|r_{p}\right|$ and ${\hat{e}}_{q}=r_{q}/\left|r_{q}\right|$.

Thus it can get:

(4) $$\begin{array}{cc} s_{p,q}\left(t\right)\cdot s_{ref}\left(t\right)= & \;\int \int \;\sigma \left(x_{T},y_{T}\right)\exp\left(j2\pi \left[f_{p}\left(t-\frac{R_{p,T}}{c}-\frac{R_{q,T}}{c}\right)\right]\right)\\ \; & \times \exp\left(-j2\pi \left[f_{p}\left(t-\frac{\left|r_{p}\right|}{c}-\frac{\left|r_{q}\right|}{c}\right)\right]\right)dx_{T}dy_{T}\\ = & \;\int \int \;\sigma \left(x_{T},y_{T}\right)\exp\left(j2\pi K_{p,q}\cdot r_{T}\right)dx_{T}dy_{T} \end{array}$$

where $K_{p,q}=\left(k_{p,q}^{x},k_{p,q}^{y}\right)$ · $k_{p,q}^{x}$ and $k_{p,q}^{y}$ are:

(5) $$\begin{array}{cc} k_{p,q}^{x} & =\frac{f_{p}}{c}\left(\cos\theta_{p}\sin\varphi_{p}+\cos\theta_{q}\sin\varphi_{q}\right)\\ k_{p,q}^{y} & =\frac{f_{p}}{c}\left(\cos\theta_{p}\cos\varphi_{p}+\cos\theta_{q}\cos\varphi_{q}\right) \end{array}$$

Therefore, the value of the 2D spatial spectral point $\left(k_{p,q}^{x},k_{p,q}^{y}\right)$ of the imaging plane is obtained:

(6) $$G\left(k_{p,q}^{x},k_{p,q}^{y}\right)=\;\int \int \;\sigma \left(x_{T},y_{T}\right)\exp\left(j2\pi \left(x_{T}k_{p,q}^{x}+y_{T}k_{p,q}^{y}\right)\right)dx_{T}dy_{T}$$

Finally, the common algorithms can be applied on spatial spectral point value to obtain the target image, such as the inverse fast Fourier transform (IFFT) algorithm, the back-projection (BP) algorithm [23], and the non-uniform fast Fourier transform [8] and so on.

### 2.2. Analysis of Model Mismatch Problem Caused by Imaging Plane Mismatch

In the actual MIMO radar imaging applications, especially in aerial target imaging, the center and the posture angles of the imaging plane are uncertain due to the target’s non-cooperative movement state. When the parameters of the imaging plane have deviations, the fulfilling region of the obtained spatial spectrum will deviate from the real region, which will cause images to be unfocused.

**Figure 2:**
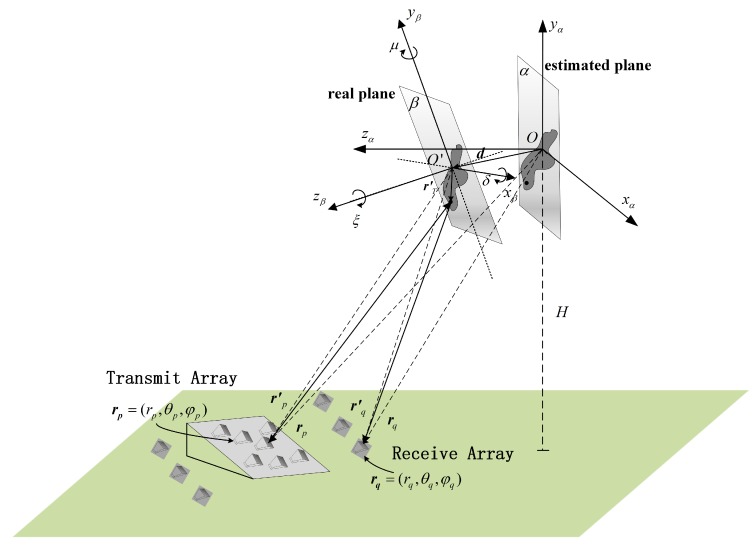
Imaging geometry of MIMO radar with imaging plane mismatch.

Figure 2 shows the imaging geometry of the scenario with imaging plane mismatch. Let α denote the estimated target plane, while let β denote the real target plane. Besides, set up the coordinate $O-x_{\alpha }y_{\alpha }z_{\alpha }$ with the origin *O* located at the center of the estimated target plane and plane $x_{\alpha }Oy_{\alpha }$ coincides with the estimated target plane. The location vectors of *p*-th transmitting antenna and *q*-th receiving antenna are $r_{p}=\left(r_{p},\theta_{p},\varphi_{p}\right)$ and $r_{q}=\left(r_{q},\theta_{q},\varphi_{q}\right)$ respectively in Coordinate $O-x_{\alpha }y_{\alpha }z_{\alpha }$. Likewise, set up the coordinate $O^{\prime }-x_{\beta }y_{\beta }z_{\beta }$ with the origin $O^{\prime }$ located at the center of the real target plane and plane $x_{\beta }Oy_{\beta }$ coincides with the real target plane. Meanwhile, the location vectors of *p*-th transmitting antenna and *q*-th receiving antenna are $r_{p}^{\prime }=\left(r_{p}^{\prime },\theta_{p}^{\prime },\varphi_{p}^{\prime }\right)$ and $r_{q}^{\prime }=\left(r_{q}^{\prime },\theta_{q}^{\prime },\varphi_{q}^{\prime }\right)$ respectively in coordinate $O^{\prime }-x_{\beta }y_{\beta }z_{\beta }$. In addition, the changes between plane β and plane α consist of the translation change and the posture angles’ change. The direction vector $d=\left(d_{\beta },\theta_{\beta },\varphi_{\beta }\right)$ represents the translation of the origin of coordinate $O^{\prime }-x_{\beta }y_{\beta }z_{\beta }$ relative to the origin of coordinate $O-x_{\alpha }y_{\alpha }z_{\alpha }$. Define (δ,μ,ξ) as the posture angle of plane β in Coordinate $O-x_{\alpha }y_{\alpha }z_{\alpha }$, where δ denotes the angle between the axis $x_{\beta }$ of coordinate $O^{\prime }-x_{\beta }y_{\beta }z_{\beta }$ and the plane $x_{\alpha }Oy_{\alpha }$, μ denotes the angle between the projection of the axis $x_{\beta }$ on the plane $x_{\alpha }Oz_{\alpha }$ and the axis $x_{\alpha }$, and ξ denotes the angle between the plane $y_{\beta }O^{\prime }z_{\beta }$ and the plane $y_{\alpha }Oz_{\alpha }$.

What is more, $\theta_{p}^{\prime },\theta_{q}^{\prime },\varphi_{p}^{\prime },\varphi_{q}^{\prime }$ and $\theta_{p},\theta_{q},\varphi_{p},\varphi_{q}$ have relations:

(7) $$\begin{array}{cc} \theta_{p}^{\prime }=\theta_{p}+\delta \sin\varphi_{p}+\mu \cos\varphi_{p},\; & \varphi_{p}^{\prime }=\varphi_{p}+\xi +\delta \cos\varphi_{p}+\mu \sin\varphi_{p}\\ \theta_{q}^{\prime }=\theta_{q}+\delta \sin\varphi_{q}+\mu \cos\varphi_{q},\; & \varphi_{q}^{\prime }=\varphi_{q}+\xi +\delta \cos\varphi_{q}+\mu \sin\varphi_{q} \end{array}$$

Hence, $r_{p}^{\prime }$ and $r_{q}^{\prime }$ can be computed by $r_{p}$, $r_{q}$ and d, namely

(8) $$\begin{array}{cc} \left|r_{p}^{\prime }\right| & =\left|r_{p}-d\right|=\sqrt{{\left|r_{p}\right|}^{2}+d_{\beta }^{2}-2\left|r_{p}\right|\cdot d_{\beta }\left(\cos\theta_{p}\cos\theta_{\beta }\cos\left(\varphi_{p}-\varphi_{\beta }\right)+\sin\theta_{p}\sin\theta_{\beta }\right)}\\ \left|r_{q}^{\prime }\right| & =\left|r_{q}-d\right|=\sqrt{{\left|r_{q}\right|}^{2}+d_{\beta }^{2}-2\left|r_{q}\right|\cdot d_{\beta }\left(\cos\theta_{q}\cos\theta_{\beta }\cos\left(\varphi_{q}-\varphi_{\beta }\right)+\sin\theta_{q}\sin\theta_{\beta }\right)} \end{array}$$

Substitute Equation (8) into Equation (3), so that it can get:

(9) $$\begin{array}{cc} \tau_{p,T}= & \frac{R_{p,T}}{c}=\frac{\left|r_{p}^{\prime }\right|+x_{T}\cos\theta_{p}^{\prime }\sin\varphi_{p}^{\prime }+y_{T}\cos\theta_{p}^{\prime }\cos\varphi_{p}^{\prime }}{c}\\ = & \frac{\sqrt{{\left|r_{p}\right|}^{2}+d_{\beta }^{2}-2\left|r_{p}\right|d_{\beta }\left(\cos\theta_{p}\cos\theta_{\beta }\cos\left(\varphi_{p}-\varphi_{\beta }\right)+\sin\theta_{p}\sin\theta_{\beta }\right)}}{c}\\ \; & +\frac{x_{T}\cos\theta_{p}^{\prime }\sin\varphi_{p}^{\prime }+y_{T}\cos\theta_{p}^{\prime }\cos\varphi_{p}^{\prime }}{c}\\ \tau_{q,T}= & \frac{R_{q,T}}{c}=\frac{\left|r_{q}^{\prime }\right|+x_{T}\cos\theta_{q}^{\prime }\sin\varphi_{q}^{\prime }+y_{T}\cos\theta_{q}^{\prime }\cos\varphi_{q}^{\prime }}{c}\\ = & \frac{\sqrt{{\left|r_{q}\right|}^{2}+d_{\beta }^{2}-2\left|r_{q}\right|d_{\beta }\left(\cos\theta_{q}\cos\theta_{\beta }\cos\left(\varphi_{q}-\varphi_{\beta }\right)+\sin\theta_{q}\sin\theta_{\beta }\right)}}{c}\\ \; & +\frac{x_{T}\cos\theta_{q}^{\prime }\sin\varphi_{q}^{\prime }+y_{T}\cos\theta_{q}^{\prime }\cos\varphi_{q}^{\prime }}{c} \end{array}$$

Since the real imaging plane parameters are not accurately known, the reference signal $s_{ref}\left(t\right)$ is still the same as Equation (2). Substitute Equation (9) into Equation (4) and using far field approximation:

(10) $$\begin{array}{cc} s_{p,q}\left(t\right)\cdot s_{ref}\left(t\right)=\;\int \int \; & \sigma \left(x_{T},y_{T}\right)\exp\{j2\pi \frac{f_{p}}{c}(-d_{\beta }\left(\cos\theta_{p}\cos\theta_{\beta }\cos\left(\varphi_{p}-\varphi_{\beta }\right)+\sin\theta_{p}\sin\theta_{\beta }\right)\\ - & d_{\beta }\left(\cos\theta_{q}\cos\theta_{\beta }\cos\left(\varphi_{q}-\varphi_{\beta }\right)+\sin\theta_{q}\sin\theta_{\beta }\right)\\ + & x_{T}[\cos\left(\theta_{p}+\delta \sin\varphi_{p}+\mu \cos\varphi_{p}\right)\sin\left(\varphi_{p}+\xi +\delta \cos\varphi_{p}+\mu \sin\varphi_{p}\right)\\ + & \cos\left(\theta_{q}+\delta \sin\varphi_{q}+\mu \cos\varphi_{q}\right)\sin\left(\varphi_{q}+\xi +\delta \cos\varphi_{q}+\mu \sin\varphi_{q}\right)]\\ + & y_{T}[\cos\left(\theta_{p}+\delta \sin\varphi_{p}+\mu \cos\varphi_{p}\right)\cos\left(\varphi_{p}+\xi +\delta \cos\varphi_{p}+\mu \sin\varphi_{p}\right)\\ + & \cos\left(\theta_{q}+\delta \sin\varphi_{q}+\mu \cos\varphi_{q}\right)\cos\left(\varphi_{q}+\xi +\delta \cos\varphi_{q}+\mu \sin\varphi_{q}\right)])\}dx_{T}dy_{T} \end{array}$$

Therefore Equation (6) actually gets:

(11) $$\begin{array}{cc} G\left(k_{p,q}^{x},k_{p,q}^{y}\right)= & \;\int \int \;\sigma \left(x_{T},y_{T}\right)\exp\{j2\pi \left(x_{T}k_{p,q}^{x}+y_{T}k_{p,q}^{y}\right)+j2\pi \frac{f_{p}}{c}(\\ - & d_{\beta }\left(\cos\theta_{p}\cos\theta_{\beta }\cos\left(\varphi_{p}-\varphi_{\beta }\right)+\sin\theta_{p}\sin\theta_{\beta }\right)\\ - & d_{\beta }\left(\cos\theta_{q}\cos\theta_{\beta }\cos\left(\varphi_{q}-\varphi_{\beta }\right)+\sin\theta_{q}\sin\theta_{\beta }\right)\\ + & x_{T}[\sin\left(\delta \sin\varphi_{p}+\mu \cos\varphi_{p}\right)\left(\sin\theta_{p}\sin\varphi_{p}+\sin\theta_{q}\sin\varphi_{q}\right)\\ + & \sin\left(\xi +\delta \cos\varphi_{q}+\mu \sin\varphi_{q}\right)\left(\cos\theta_{p}\cos\varphi_{p}+\cos\theta_{q}\cos\varphi_{q}\right)]\\ + & y_{T}[\sin\left(\delta \sin\varphi_{p}+\mu \cos\varphi_{p}\right)\left(\sin\theta_{p}\cos\varphi_{p}+\sin\theta_{q}\cos\varphi_{q}\right)\\ + & \sin\left(\xi +\delta \cos\varphi_{q}+\mu \sin\varphi_{q}\right)\left(\cos\theta_{p}\sin\varphi_{p}+\cos\theta_{q}\sin\varphi_{q}\right)])\}dx_{T}dy_{T} \end{array}$$

The location of space spectral points obtained by the above processing is not completely matched with the real spatial spectral fulfilling region. As a result, the entire image is not focused on the real target plane.

### 2.3. The Calibration Operation

In order to obtain more accurate imaging results, it is necessary to search out the parameters of the imaging plane, so as to eliminate the mismatch between the estimated spatial spectral fulfilling region and the real spatial spectral fulfilling region, and the according spatial spectral values. After the parameters of the imaging plane are obtained, the following calibration operation can be applied to achieve focused image.

When the parameters $d=\left(d_{\beta },\theta_{\beta },\varphi_{\beta }\right)$ and (δ,μ,ξ) have been obtained, the reference signal can be calibrated as:

(12) $$s_{ref}^{\prime }\left(t,r_{p}^{\prime },r_{q}^{\prime }\right)=\exp\left\{-j2\pi f_{p}\left(t-\frac{\left|r_{p}^{\prime }\right|+\left|r_{q}^{\prime }\right|}{c}\right)\right\}$$

After the coherent processing in Equation (10), the final spatial spectral value is:

(13) $$G^{\prime }\left(k_{p,q}^{{}^{\prime }x},k_{p,q}^{{}^{\prime }y}\right)=\underset{T}{\;\int \int \;}\sigma \left(x_{T},y_{T}\right)e^{j2\pi \left(x_{T}k_{p,q}^{{}^{\prime }x}+y_{T}k_{p,q}^{{}^{\prime }y}\right)}dx_{T}dy_{T}$$

where *T* denotes the imaging area and $k_{p,q}^{{}^{\prime }x}$, $k_{p,q}^{{}^{\prime }y}$ are defined in Equation (14).

(14) $$\begin{array}{cc} k_{p,q}^{{}^{\prime }x} & =\frac{f_{p}\left(\cos\theta_{p}^{\prime }\sin\varphi_{p}^{\prime }+\cos\theta_{q}^{\prime }\sin\varphi_{q}^{\prime }\right)}{c}\\ & =\frac{f_{p}}{c}\left(\left(\cos\left(\theta_{p}+\delta \sin\varphi_{p}+\mu \cos\varphi_{p}\right)\sin\left(\varphi_{p}+\xi +\delta \cos\varphi_{p}+\mu \sin\varphi_{p}\right)\right.\right.\\ & +\cos\left(\theta_{q}+\delta \sin\varphi_{q}+\mu \cos\varphi_{q}\right)\sin\left(\varphi_{q}+\xi +\delta \cos\varphi_{q}+\mu \sin\varphi_{q}\right))\\ k_{p,q}^{{}^{\prime }y} & =\frac{f_{p}\left(\cos\theta_{p}^{\prime }\cos\varphi_{p}^{\prime }+\cos\theta_{q}^{\prime }\cos\varphi_{q}^{\prime }\right)}{c}\\ & =\frac{f_{p}}{c}\left(\cos\left(\theta_{p}+\delta \sin\varphi_{p}+\mu \cos\varphi_{p}\right)\cos\left(\varphi_{p}+\xi +\delta \cos\varphi_{p}+\mu \sin\varphi_{p}\right)\right.\\ & +\cos\left(\theta_{q}+\delta \sin\varphi_{q}+\mu \cos\varphi_{q}\right)\cos\left(\varphi_{q}+\xi +\delta \cos\varphi_{q}+\mu \sin\varphi_{q}\right)) \end{array}$$

Ultimately, the focused images can be obtained using $k_{p,q}^{{}^{\prime }x}$, $k_{p,q}^{{}^{\prime }y}$ and the corresponding spatial spectral value $G^{\prime }\left(k_{p,q}^{{}^{\prime }x},k_{p,q}^{{}^{\prime }y}\right)$.

## 3. The Proposed Imaging Plane Calibration Algorithm (IPCA)

When there are deviations between the estimated values of the imaging plane parameters $\left(d_{\beta },\theta_{\beta },\varphi_{\beta },\delta ,\mu ,\xi \right)$ and the real values, it will lead to the problem of spatial spectrum mismatch. The mismatch of spatial spectrum results in the image not focusing at the real imaging plane and the whole image quality is deteriorated seriously.

In order to achieve better imaging performance, it is necessary to search out the real imaging plane parameters, namely the six parameters $\left(d_{\beta },\theta_{\beta },\varphi_{\beta },\delta ,\mu ,\xi \right)$.

However, searching directions cannot be clear at beginning and always changed in the processing. Commonly, a good objective function will make the searching process in desirable directions.

Generally, image entropy can be used to measure the focusing performance of an image. In radar imaging, it is defined as follows:

(15) $$E=-\sum_{k=0}^{M-1}\sum_{n=0}^{N-1}\frac{{\left|\sigma \left(x_{k},y_{n}\right)\right|}^{2}}{S}\ln\frac{{\left|\sigma \left(x_{k},y_{n}\right)\right|}^{2}}{S}$$

where, $S=\sum_{k=0}^{M-1}\sum_{n=0}^{N-1}{\left|\sigma \left(x_{k},y_{n}\right)\right|}^{2}$, and the image is discretized into M×N grids. $\sigma (x_{k},y_{n})$ denotes the scattering coefficient of the grid in *k*-th row at *n*-th column. At the same time, in the aerial imaging applications, the targets usually have sparse characteristics.

Therefore, both the focusing performance and the sparsity of the targets are considered, hence the objective function is to minimize the following function:

(16) $$f\left(d,\delta ,\mu ,\xi \right)=-\sum_{k=0}^{M-1}\sum_{n=0}^{N-1}\frac{{\left|\sigma \left(x_{k},y_{n}\right)\right|}^{2}}{S}\ln\frac{{\left|\sigma \left(x_{k},y_{n}\right)\right|}^{2}}{S}+\gamma \sum_{k=0}^{M-1}\sum_{n=0}^{N-1}{\left|\sigma \left(x_{k},y_{n}\right)\right|}_{1}$$

where $\sigma \left(x_{k},y_{n}\right)=IFFT\left(G_{p,q}\left(k_{x},k_{y},d,\delta ,\mu ,\xi \right)\right)$ and γ is a tunable parameter to adjust the weights of the image entropy and the sparsity.

In such a way, the estimation problem for (d,δ,μ,ξ) can be transformed into the following optimization problem:

(17) $$\begin{array}{cc} \left(d,\delta ,\mu ,\xi \right) & =\underset{d,\delta ,\mu ,\xi }{\arg\min}f\left(d,\delta ,\mu ,\xi \right)\\ s.t.\;\sigma \left(x_{k},y_{n}\right) & =\mathrm{IFFT}\left(G_{p,q}\left(k_{x},k_{y},d,\delta ,\mu ,\xi \right)\right) \end{array}$$

However, the above optimization problem is not a convex problem. Commonly, among non-convex optimization algorithms, metaheuristic optimization [24] is becoming increasingly popular. Particle swarm optimization (PSO) algorithm is one of the most popular metaheuristic optimization algorithms, and has been successfully applied in radar imaging problems [25,26]. Hence, the imaging plane calibration algorithm (IPCA) is proposed to utilize PSO algorithm for solving the optimization problem in Equation (17). In IPCA, each particle $p_{i}=\left(d_{\beta i},\theta_{\beta i},\varphi_{\beta i},\delta_{i},\mu_{i},\xi_{i}\right)$ denotes one estimation of $\left(d_{\beta },\theta_{\beta },\varphi_{\beta },\delta ,\mu ,\xi \right)$, while the velocity $v_{i}$ of each particle denotes one search direction. In PSO, each particle remembers its personal best position. Meanwhile, global best position is also recorded. In each iteration, the velocity of each particle is updated combining the individual movement states and the group movement states. Particles acquire their new positions by constantly updating their speed, and eventually all particles will converge to the global optimal value [20,21].

**Figure 3:**
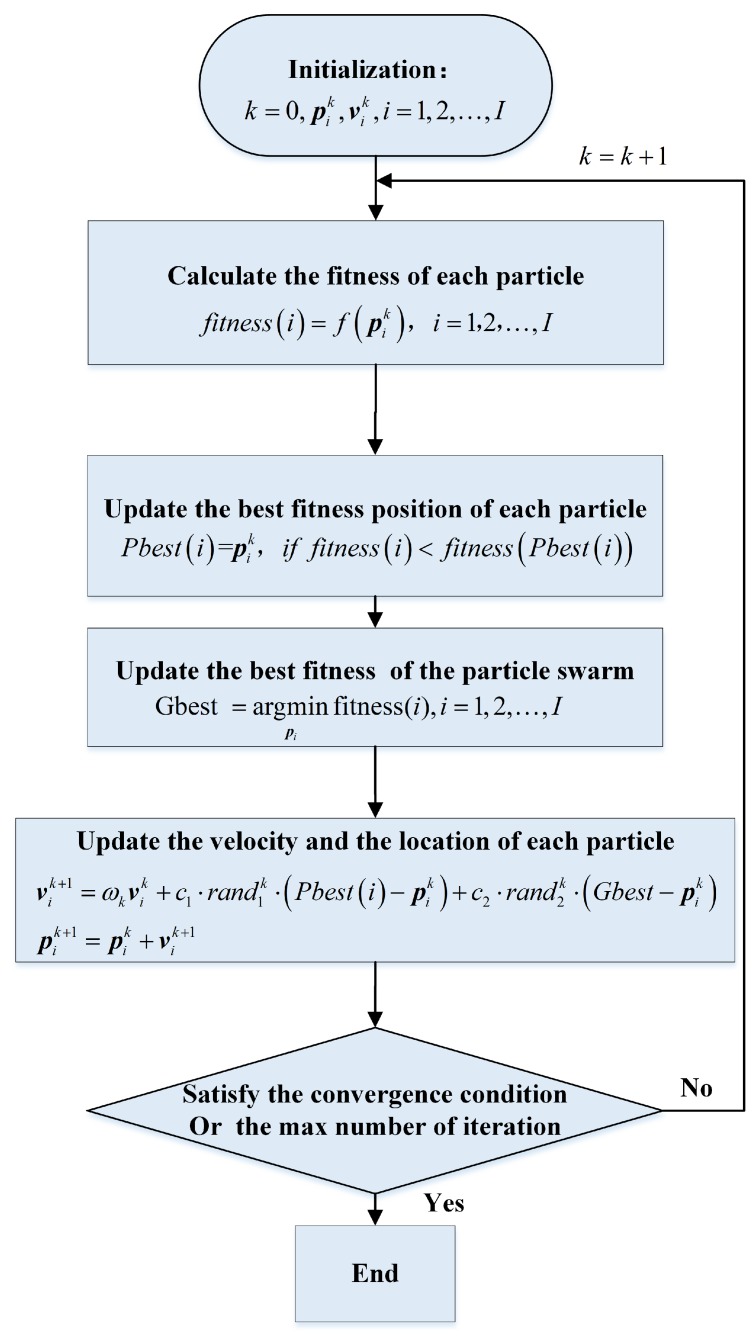
Flow chart of imaging plane calibration algorithm (IPCA).

The detailed algorithm of IPCA is described in Algorithm 1 and the flow chart is illustrated in Figure 3.

**Algorithm 1:** IPCA

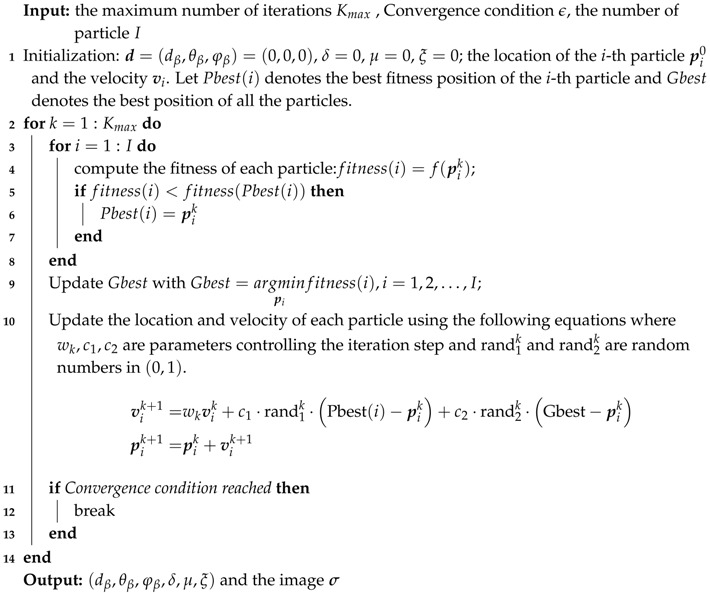



## 4. Simulations

In this section, several simulations are carried out to verify the effectiveness of IPCA.

**Figure 4:**
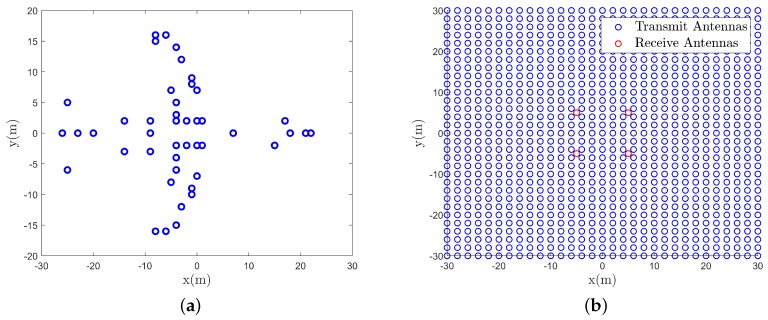
Target and antenna array (**a**) Scattering points distribution of the B727 airplane, (**b**) Locations of transmit antennas and receive antennas.

The imaging distance is set to 1 km in the simulations and the target is a B727 airplane with its point scattering model shown in Figure 4a. The size of the imaging plane, approximately parallel to the radar antenna array, is 60×60 m. The radar consists of 31×31 transmitters and 4 receivers and all of the antennas are located on the same transceiver plane, as is illustrated in Figure 4b. Besides, the size of the radar array is 60×60 m and the radar works at C-band. The detailed simulation parameters are given in Table 1.

### 4.1. Imaging Simulation

To verify the effectiveness of the proposed method, the following simulation was carried out. System parameters in the simulations are shown in Table 1. The parameters of the center and pose angles of the imaging plane both have errors. The real parameters are given in Table 2.

**Figure 5:**
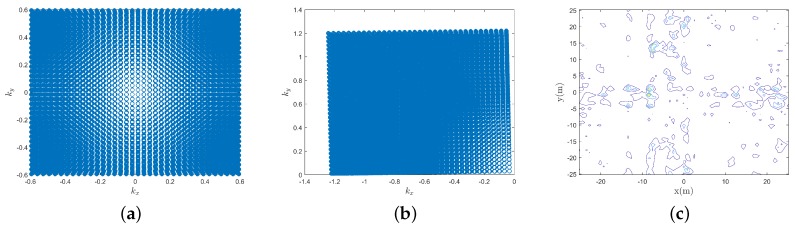
Spatial spectrum and reconstructed image. (**a**) The estimated fulfilled region of the spatial spectrum, (**b**) The real fulfilled region of the spatial spectrum, (**c**) Image reconstructed using estimated spatial spectrum values.

According to the derivation in Section 2 and the deviation parameters of the imaging plane, the estimated and real fulfilling region of the spatial spectrum are illustrated in Figure 5a,b respectively. It can be seen that when there are deviations of the imaging plane parameters, the estimated fulfilling region of the spatial spectrum is quite different from the fulfilling region of the real spatial spectrum. The recovered image is illustrated in Figure 5c when deviations of the imaging plane parameters are not known, and the adopted algorithm is the IFFT algorithm. It can been seen that even when there are slight deviations of the imaging plane parameters, e.g., the deviations of the posture angles of the imaging plane are within $1^{\circ }$, the image is badly defocused, and the target’s contour is not clear.

In the following, the proposed IPCA is taken to search out the six parameters, namely $\left(d_{\beta },\theta_{\beta },\varphi_{\beta },\delta ,\mu ,\xi \right)$, and then the calibration operation is taken to obtain the final image. The number of the particles, *I*, in IPCA is set to 100 and the maximum number of iterations, $K_{max}$, is set to 100 too. Meanwhile some other parameters in PSO are set: $\omega_{k}=0.8-0.5\times \frac{k}{K_{max}}$, $c_{1}=c_{2}=1$.

**Figure 6:**
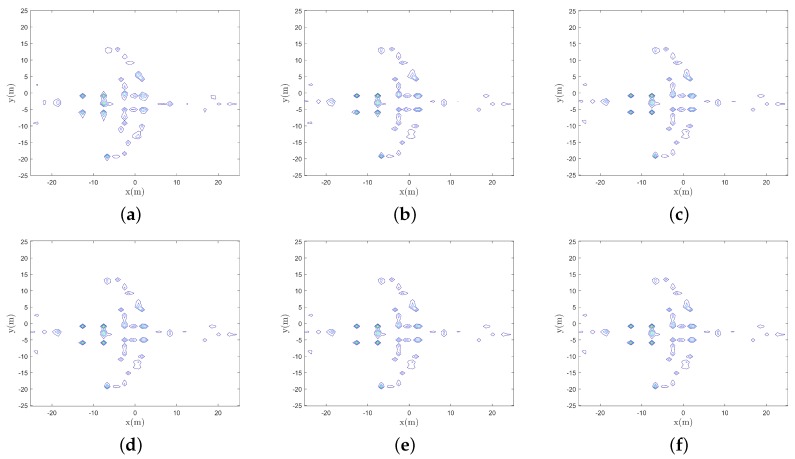
Reconstructed images of different iterations, (**a**–**f**) images of the 10th, 20th, 30th, 50th, 80th, and 100th iteration respectively.

According to Figure 6, the image has clearer target outlines and better focusing performance with fewer noisy points around the target. In the final image, each scattering point of the target can be clearly identified. Meanwhile, the image entropy is smaller using IPCA than that using IFFT, as is shown in Table 3, which means better focusing performance.

**Figure 7:**
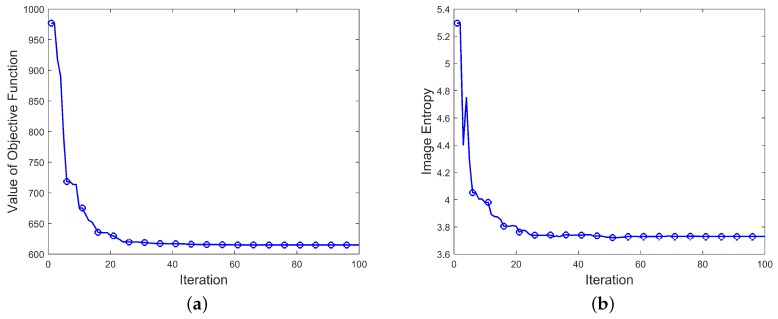
Value of objective function and image entropy during iterations. (**a**) Value of objective function during iterations (**b**) Image entropy during iterations.

Figure 7 shows the value of the objective function and the image entropy during the IPCA iterations. It can been seen that when the number of iterations exceeds 80, the value of the objective function tends to be stable, that is, the search results gradually converge.

From what has been discussed above, the method proposed in this paper can effectively converge. Meanwhile, the entropy of the inversion image is lower and the image quality is higher.

**Figure 8:**
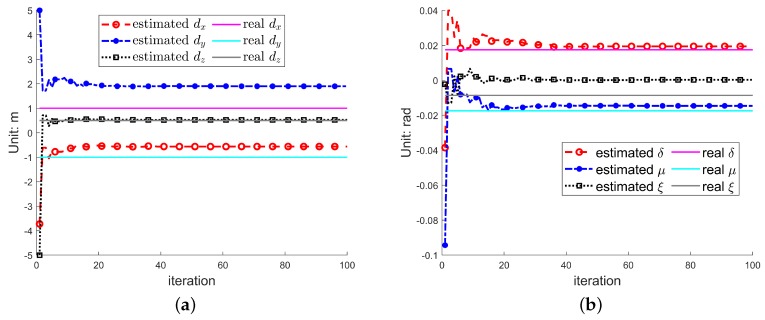
Values of $d=(d_{x},d_{y},d_{z})$ and (δ,μ,ξ) during iterations. (**a**) $d=(d_{x},d_{y},d_{z})$, (**b**) (δ,μ,ξ).

Figure 8a shows the estimated $d=(d_{x},d_{y},d_{z})$ during the iterations. It can be seen that, with the increase of the number of iterations, $d_{z}$ gradually approaches the real value, and finally converges to the real value. The values of $d_{x}$$d_{y}$ are still deviated from the real values. This is because the deviations $d_{x}$, $d_{y}$ of the image will only make the image translation in the imaging plane, which have no effect on the image focus effect and target sparsity. Therefore, $d_{x}$, $d_{y}$ do not affect the imaging quality and IPCA cannot guarantee $d_{x}$, $d_{y}$ convergence to the real value. Likewise, Figure 8b shows the estimated imaging plane posture angles (δ,μ,ξ) with the number of iterations. It can be seen that as the number of iterations increases, the value of δ,μ converges to the real value while ξ not. This is because the estimation error of the rotation angle ξ, whose rotating axis parallel to the line of sight, will make the image rotate in the imaging plane, which has no influence on the image entropy and target sparsity. Therefore ξ does not affect the imaging quality and IPCA cannot guarantee ξ convergence to the real value.

Meanwhile, since the PSO algorithm is a metaheuristic optimization algorithm, the computation time of IPCA is hard to predict. Therefore, 10 Monte Carlo trials are taken to count the computation time. The computation times of 10 Monte Carlo trials vary from 954 s to 2017 s with average computation time is 1617 s.

To sum up, the algorithm proposed in this paper can obtain more accurate imaging plane parameters, resulting in better image focusing performance and better imaging quality.

### 4.2. Simulations with Different Tunable Parameter γ

In Equation (16), a tunable parameter γ is defined to adjust the weights of the image entropy and the sparsity. In order to analysis the influence of γ, the below simulations are taken.

**Figure 9:**
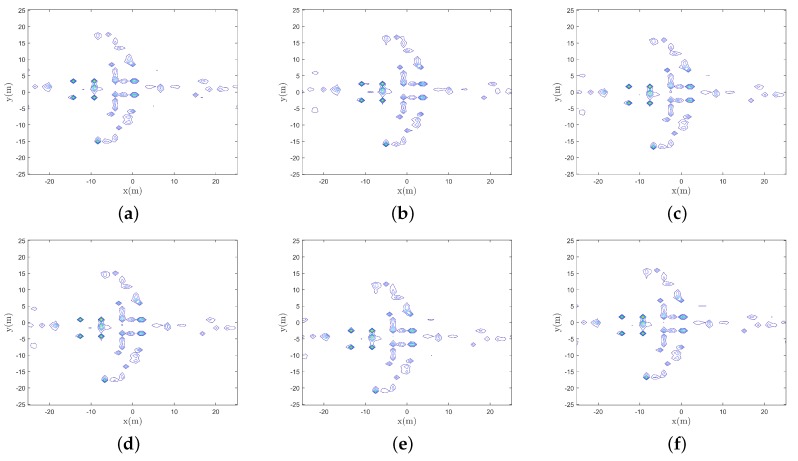
Reconstructed images with different γ, (**a**) γ=0.1, (**b**) γ=1, (**c**) γ=3, (**d**) γ=5, (**e**) γ=10, (**f**) γ=50.

The simulation parameters are set the same with that in Table 1 and Table 2. γ is set to [0.1,0.5,1,3,5,10,50]. The imaging results are illustrated in Figure 9, meanwhile the image entropy are given in Table 4.

It can be seen from Figure 9 that the reconstructed images are all well focused and the image entropy are all lower than 4 when γ is chosen with different values. So it can conclude that γ has seldom influence on the final imaging results. Therefore, γ can be selected from a wide range in IPCA.

### 4.3. Simulations under Different Signal-to-Noise Ratios (SNRs)

**Figure 10:**
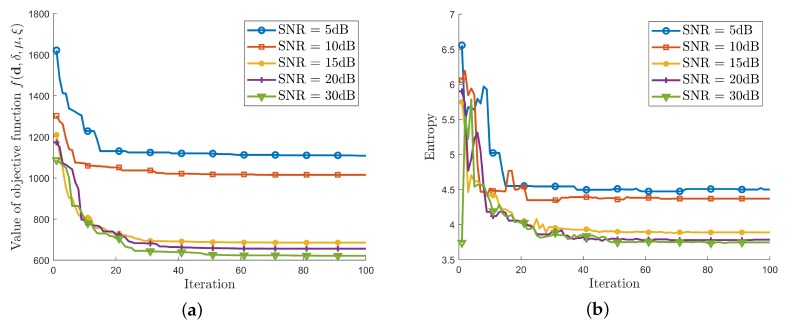
(**a**) Value of the objective function f(d,δ,μ,ξ) during iterations under different SNRs, (**b**) Image entropy during iterations under different SNRs.

In order to verify the robustness to the noise of IPCA, the following simulation is carried out in different echo signal-to-noise ratio. The echo SNRs are set from 5 dB to 30 dB. The simulation parameters are the same with that in Table 1 and Table 2. The simulation results are in Figure 10.

The value of the objective function f(d,δ,μ,ξ) during the iterations is illustrated in Figure 10a. As it is shown, the value of the objective function f(d,δ,μ,ξ) decreases iteratively and finally converges. When the SNR is above 15 dB, the final values of f(d,δ,μ,ξ) are closed to each other.

The image entropy during the iterations is illustrated in Figure 10b. It can be seen that image entropy decreases iteratively. When the SNR is equal to or above 15 dB, the final image entropy is lower than 4 which means good image focusing performance.

### 4.4. Simulations under Different Parameter Ranges

In order to verify that the proposed method has a certain degree of tolerance for the deviations of the imaging parameters, the following simulations are conducted. According to the first subsection in this section, the center deviation parameter $d_{x}$, $d_{y}$ of the imaging plane only cause the image to shift in the imaging plane, and has no influence on the imaging quality and focusing performance. In addition, the posture angle ξ makes the image rotate in the imaging plane, while the focusing performance is not affected. So in the following simulations, only $d_{z}$, δ and μ are considered.

In the simulations, $d_{z}$, δ and μ are uniform random selected in $\left[-\frac{\Delta d_{z}}{2},\frac{\Delta d_{z}}{2}\right]$, $\left[-\frac{\Delta \delta }{2},\frac{\Delta \delta }{2}\right]$ and $\left[-\frac{\Delta \mu }{2},\frac{\Delta \mu }{2}\right]$. $\Delta d_{z}$ are set to [0.1,0.2,0.3,0.5,0.8,1.0], while Δδ are set to [1,2,3,5,8,10]/180π whereas Δμ are set to [1,2,3,5,8,10]/180π. For each $\Delta d_{z}$, Δδ and Δμ, 10 Monte Carlo trials are taken. Meanwhile, when the value $d_{z}$, δ and μ are changing, $d_{x}$, $d_{y}$ and ξ are set to be zeros.

**Figure 11:**
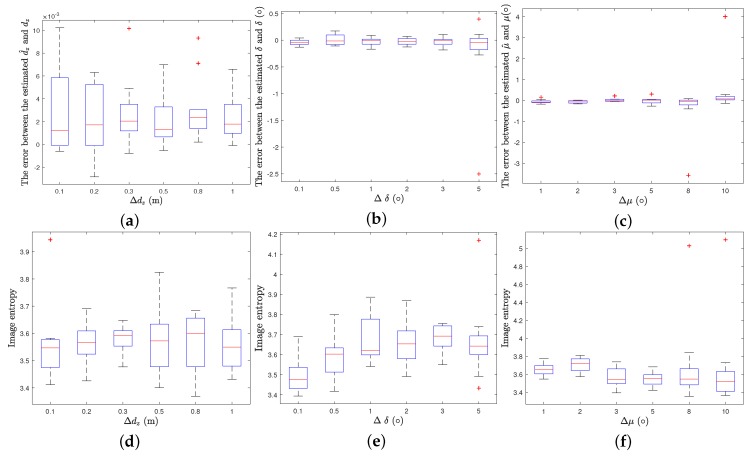
Errors between the estimated $d_{z}$, δ, μ and real values and Image entropy of 10 Monte Carlo trials, (**a**–**c**). Errors between the estimated $d_{z}$, δ, μ and real values respectively when different $\Delta d_{z}$, Δδ and Δμ are chosen; (**d**–**f**) image entropy with different $\Delta d_{z}$, Δδ and Δμ.

The errors between the estimated $d_{z}$, δ, μ and the real values and the image entropy obtained by 10 Monte Carlo trials with different $\Delta d_{z}$, Δδ, Δμ are illustrated using boxplot in Figure 11. On each box, the central mark indicates the median, and the bottom and top edges of the box indicate the 25th and 75th percentiles, respectively. The whiskers extend to the most extreme data points not considered outliers, and the outliers are plotted individually using the red ’+’ symbol. According to Figure 11a–c, the errors between the final estimated $d_{z}$ and the real values are within ±0.01 m, and the errors between the final estimated δ and the real value are within $\pm 0.35^{\circ }$ when Δδ is chosen little than $3^{\circ }$. Moreover the errors between the final estimated μ and the real values are within $\pm 0.35^{\circ }$ when Δμ is chosen little than $5^{\circ }$. In addition, according to Figure 11d–f, the image entropy are all lower than 4 when $\Delta d_{z}$, Δδ, Δμ are chosen within 1 m, $3^{\circ }$, $5^{\circ }$ respectively.

In short, IPCA can obtain the final estimated $d_{z}$, δ and μ with errors at most ±0.01 m, $\pm 0.35^{\circ }$ and $\pm 0.35^{\circ }$ when $\Delta d_{z}$, Δδ, Δμ are chosen within 1 m, $3^{\circ }$, $5^{\circ }$ respectively, and the image entropy is smaller than 4.

## 5. Conclusions

In this paper, the imaging plane mismatch problem of 2D cross-range MIMO radar imaging is analyzed, and the deviations between the estimated spatial spectral point location and the real spatial spectral point location are deduced as well as the corresponding spatial spectral values. To solve this problem, IPCA is proposed in this paper. Aiming at minimizing the image entropy and sparsity of the image, PSO is utilized in IPCA to obtain the image with better focusing performance. Simulation results verify the effectiveness of the proposed algorithm and the robustness to noise. At the same time, when the parameters of the imaging plane are different, the proposed algorithm can obtain the imaging plane parameters of the real values, and then carry out the imaging plane calibration operation to obtain the focused image. The method proposed in this paper can solve the imaging plane mismatch problem and obtain high quality MIMO images.

## Figures and Tables

**Table 1:** Simulation parameters.

Parameter	Value
Imaging distance	1 km
The size of the imaging plane	60 m ×60 m
The size of the radar antenna array	60 m ×60 m
Number of transmitters	31×31
Number of receivers	4
Carrier frequency	5 GHz
Bandwidth	200 MHz

**Table 2:** Imaging plane parameters.

Parameter	Value
Deviation of the center of the imaging plane	$d=\left(d_{\beta },\theta_{\beta },\varphi_{\beta }\right)=\left(1.5\;m,-0.785\;\mathrm{rad},0.340\;\mathrm{rad}\right)$
Deviation of the posture angle of the imaging plane	$\left(\delta ,\mu ,\xi \right)=\left(\frac{1}{180}\pi ,-\frac{1}{180}\pi ,-\frac{1}{180}\pi \right)\;\mathrm{rad}$

**Table 3:** Image entropy.

Adopted Algorithm	IFFT	The Proposed IPCA
Image entropy	5.66	3.729

**Table 4:** Image entropy with different γ.

γ	0.1	0.5	1	3	5	10	50
Image entropy	3.76	3.70	3.63	3.73	3.73	3.74	3.75
